# Applying for a sub-specialty fellowship: some tips and advice from a former program director

**DOI:** 10.3402/jchimp.v1i3.8087

**Published:** 2011-10-17

**Authors:** Richard M. Pomerantz

**Affiliations:** Department of Medicine, St. Agnes Hospital, Baltimore, MD, USA

**Keywords:** internal medicine, fellowship, application

## Abstract

Successfully completing an internal medicine residency is a great accomplishment. With the great need for primary care physicians, many residents are considering entering the field of general medicine where one can care for the whole patient. However, for those who have a drive to become an expert in one particular organ system, the path to the completion of training is only half over. Though you may think you have figured out the ‘application process’ after successfully gaining admission to college, medical school, and residency, there is one more hurdle that stands between you and your ultimate goal of sub-specialization – the fellowship application process. Unfortunately, this last application may be the most difficult of all. As a former cardiology program director, I have trained over 100 fellows and reviewed over 3,000 applications. Here is some practical advice from the ‘inside’ on how to be successful at this process.

Choosing a specialty is perhaps the most important choice of your young medical career. It is what you will most likely be doing on a daily basis even 30 or 40 years from now. Before choosing a specialty, try to get some experience during residency or medical school in that specialty. Don't just spend time in the hospital with a specialist. Go to his or her office and get a good feeling for what they do all day. Is it something that will interest you and keep you stimulated 20 years from now or will it be a boring grind? Consider your options carefully before you take the plunge.

If you have an academic career interest, get involved in research in the field. Meaningful research will likely result in an abstract or manuscript at the conclusion of the research project, and these publications will strengthen your application.

## Building a favorable application

Spending a year as a chief resident is also very worthwhile, as it is a sign of both seasoning and maturity and is likely to be viewed favorably for any fellowship candidate. The fellows I have trained who have done a chief residency are invariably some of the most mature and successful trainees with whom I have worked.

On the flip side, spending a year or two as a hospitalist or moonlighter to work off some debt may not be optimal. Be aware that the longer you are away from training, the less desirable candidate you may become. A maximum of a year or two doing this type of work is a realistic limit before it hurts your chances. Also, try to find a research project in your area of true interest. Nothing is worse in an interview as a candidate who has little interest in their research and has merely done it to ‘buff up’ their application.

## Deciding where to apply

Study the websites of the various training programs, ask colleagues and mentors about programs they recommend, and contact people from your residency program that are already training at the institutions where you have interest. You should come up with a reasonable list but unless you have a strong aversion, always apply to your home program as well. Your chances at your own program are likely better than elsewhere, especially if you have been a ‘star’ in your residency program.

Make sure you understand all the requirements of the application process at a particular institution including visa status. Don't bother to apply if your visa status is not accepted at the institution (many academic centers do not accept H1B visas). I could never even consider these applications and applying was really a complete waste of everyone's time.

## The application process

Carefully plan out what you want to say in the application. Since these are now done electronically, you only need submit one so put some time and effort into it. Get started early. Note that the specialty match timing has changed (in my opinion for the better) for candidates who wish to start fellowships in July 2013. The match will open in August of your third year of residency (August 2012), the interview process will probably occur through the late summer and fall and match day will be in early December 2012. This will allow you more time to decide on the right specialty, spend more time on electives, do research, and develop relationships with mentors and specialists who may provide recommendations for *you*. Though most programs have a deadline, many will start looking at applications as they come in. You stand a better chance for your application to get a better look before the program director or admissions committee has seen over 400 of them. I always worried about the time management skills of people who submitted applications on the last day by FedEx.

Remember to be completely truthful. If you did some significant research, state it, but don't overplay your involvement. You will be asked about it. If you have a limited and superficial knowledge of the subject, it will be very clear to the interviewer. Remember, you are often talking to an expert in the field. I remember one conversation with an applicant where he wasn't even pronouncing the name of the disease correctly that he supposedly had been doing research on for the past 2 years. Clearly, he had little or no real involvement in the project.

Also, it pays to note your outside interests aside from medicine. I was always interested in learning whether we were considering a normal, well-rounded individual. Things like hobbies, sports, music, and other interests and special life experiences often showed me whether this would be a nice person to have on our team. The purpose of the essay is to tell a bit about you. Lecturing me on the future of cardiology or the ‘great advances’ that have come over the past few years gets very boring after the 400th essay. Keep it personal, interesting, and short. Think of it like a TV commercial. You only have a few moments to capture the audience's attention.

References should be from people who know you well, not just a ‘big name’ specialist with whom you had little real contact. Those letters usually read something like ‘*Dear Program Director, Resident X came to teaching rounds twice with me and answered one of my silly questions correctly. I have no real idea who he is, but I am a big name in the field, and he begged me to write him a letter*’ (or something to that effect). I have gotten too many of those types of letters in the past. Avoid the temptation unless they really know you well. It is much better to have a letter from a lesser known specialist or your internal medicine program director that says, ‘*I worked with Resident X extensively on the wards, in the ICU, at night, and on call. He is fantastic and is someone who I would not hesitate to have care for any one of my family members. He is hard working, reliable, always upbeat and cheerful, and a great team player. We will be recruiting him heavily to remain here in our fellowship program*’.

## The interview

You can call once to make sure your application is complete, but do not call more than that. Don't ask if you are being considered for an interview. The secretary won't know and you will be contacted by the program if they want to meet with you. Don't be a pest; it can actually work against you. When contacted for an interview, call back quickly and pick one of the dates being offered. If you have to change the date or cancel, do so as far in advance as possible. Canceling an interview shortly before it is scheduled is not fair to the program or to the other applicants. It is too late to fill one of these coveted interview spots and often the program coordinator has done a lot of work scheduling your interviews with the faculty. Even if you don't think you will attend that institution for fellowship, the academic world is small and one day you may be asking that same program director for a job. They may remember if you did something rude and thoughtless when applying for fellowship.

Plan your trip to arrive the night before, so there is no problem with travel delays. Sleep well the night before and show up on time. Being late for the interview day is disruptive and speaks poorly to your reliability. Dress professionally and be courteous to everyone. The program director knows his coordinator and secretaries a lot longer than he has known you, so if you are discourteous, arrogant, or condescending to the administrative staff it will definitely get back to the program director! The reaction of the administrative staff can be ‘the kiss of death’ for your application if you make a negative impression. My program coordinator always told me who was nasty, condescending, or arrogant when I was out of earshot and that told me volumes about their real character. So even if you are nasty, condescending, or arrogant, hide it well from everyone that day!

Sit up straight, make eye contact with the interviewer, and be prepared and enthusiastic. I have had interviewees who literally looked at their shoes the entire time and never looked me in the eye or would slump in their chairs and asked me things like ‘so why should I come here?’ I smiled but was secretly thinking ‘don't worry, no chance of that happening!’

Think ahead about an interesting case in the field as you may be asked to present something you have seen. I have had people who have been through 4 years of medical school and 2 years of residency who couldn't think of a single interesting case to tell me about! If you did research, state what you did in the project clearly and succinctly. Understand the background and details of the research. As I mentioned, you may be talking to an expert and you can't ‘wing it’ as they will find you out. Have some pertinent questions about the program ready and know clearly why you are applying there. You may ask a few logistical questions about things like on call schedules, but don't dwell too much on the work aspects of the fellowship. It may send a red flag to both the faculty (and the current fellows) that you don't want to work hard and might be a complainer. Of all the headaches for a fellowship director, the chronic complainers are by far the worst. Obviously this is not the impression you want to make.

Writing thank you notes afterward is a nice touch but be sure to personalize them. I have gotten so many ‘form’ thank you notes that most just go right in the trash. Sometimes I have even gotten ones addressed to other program directors by mistake!

## The match

Think carefully about where you want to train. Take family considerations into account as both you and your family must be reasonably content for the duration of your fellowship. Family strife, an unhappy spouse and kids, or long distance separations can make concentrating on your training nearly impossible. However, make sure there is some permanence to the relationship before making a major career decision. I have known residents who picked a program they didn't particularly like in a certain city just because their girlfriend/boyfriend or fiancée was there and then they broke up before even starting their fellowship! You are then left with an undesirable program and a broken personal life. Not a great way to start a new chapter in your life. Definitely take into account where you will live, but remember trivial things like the weather, city amenities, and so on shouldn't greatly decide where you train. You will only be there for 2 to 3 years, and where you train has a tremendous effect on your future career for the next 40!

Some programs continue to accept some candidates outside the match. If you are in a very competitive field (like cardiology) you may want to consider taking a spot if it is outside the match and offered to you. This is an individual decision that needs to be weighed based on the competitiveness of the field, the program's desirability, and the strength of your application.

Rank your programs based on their desirability, not where you think you will get in. The match process will take care of that. I also never thought there was a great need to call the program director to express interest. The match actually discourages this practice. There is no need to tell the program director that they are your ‘top choice’. If you do, call only the program you are most interested in and don't have anyone else call for you (like your internal medicine program director) unless that truly is your top choice. I received a call once from a program director just 2 days before the match list went in, who told me in the clearest possible terms how we were the applicant's number one choice and how much she really wanted to come to our program. She was a great candidate and I had already ranked her high enough to match with us. When she didn't match, I called the program director. He felt very silly for being misled by one of his residents for whom he had done a favor. Remember, medicine is a small world and these lapses in truthfulness could eventually come back to hurt you someday.

Hopefully these inside tips will be useful as you start the fellowship application process. Certainly put your best foot forward, but always be yourself and be truthful. After many years of ‘the game’, most program directors have a sense for the ‘good ones’ out there.

